# A School-Based Environmental Intervention to Reduce Smoking among High School Students: The Acadiana Coalition of Teens against Tobacco (ACTT)

**DOI:** 10.3390/ijerph6041298

**Published:** 2009-03-27

**Authors:** Carolyn C. Johnson, Leann Myers, Larry S. Webber, Neil W. Boris, Hao He, Dixye Brewer

**Affiliations:** 1 Department of Community Health Sciences, Tulane University School of Public Health and Tropical Medicine / 1440 Canal Street, Suite 2301, New Orleans, Louisiana, 70112, USA; 2 Department of Biostatistics, Tulane University School of Public Health and Tropical Medicine / 1440 Canal Street, Suite 2001, New Orleans, Louisiana, 70112, USA; E-Mails: myersl@tulane.edu (L.M.); lswebber@tulane.edu (L.S.W.); hhe@tulane.edu (H.H.); 3 Department of Psychiatry and Neurology, Tulane University School of Medicine / 1440 Canal Street, New Orleans, Louisiana, 70112, USA; E-Mail: nboris@tulane.edu; 4 Tangipahoa Parish Health Unit, 15481 Club Deluxe Road, Hammond, Louisiana, 70403, USA; E-Mail: dixyelsu1972@aol.com

**Keywords:** Smoking prevalence, adolescents, high school, health promotion

## Abstract

A school-based environmental program to reduce adolescent smoking was conducted in 20 schools (10 intervention; 10 control) in south central Louisiana. The 9^th^ grade cohort (n = 4,763; mean age = 15.4 yrs; 51% female; 61% Caucasian; 30-day smoking prevalence at baseline = 25%) was followed over four years for 30-day smoking prevalence with the school as the unit of analysis. Although prevalence decreased in intervention schools and increased in control schools in Year 2 the significant difference between the two groups at baseline was not overcome by the intervention and increases in prevalence were observed in both groups in Years 3 and 4. The higher the percentage of white students in a school the higher the prevalence rates regardless of intervention/control status. Boys’ and girls’ smoking rates were similar. These outcome data, student feedback and process evaluation provide a basis for continuing to create more innovative adolescent tobacco control programs.

## 1. Introduction

In the early part of the 1990s, national data tracked alarming increases in adolescent smoking [1–3]. By the mid-1990’s, adolescent smoking rates were the highest that had been recorded in a quarter of a century. By 1996–97, teen smoking began to decrease by slight percentages, and it was felt that the prevalence would continue to decline [4]; nevertheless, the trends observed both nationally and statewide were alarming in view of the fact that individuals who adopted risky behaviors in adolescence tended to persist in those behaviors as adults, representing a large segment of the population with a high potential for illness and disease in the not-to-distant future. Estimates showed that if children who were smoking then continued to smoke as adults, more than five million would die of smoking-related diseases [5]. The more immediate problems that youth can experience from smoking have been identified as chronic respiratory problems, early development of cardiovascular risk, and many other health implications of initiating use at young ages [6]. Smoking also clusters with other risk behaviors, such as alcohol and illegal drug use, sensation-seeking, violence, inappropriate low weight maintenance, and poor academic performance [7–12]. At about the same time that adolescent smoking rates were reaching their zenith, the tobacco Master Settlement Agreement (MSA) had been concluded and states now had money to contribute to prevention programs [13]. Additionally, Healthy People 2000 and 2010 stressed the importance of reducing initiation of cigarette smoking by youth [5,14].

Although reported rates for teenage smoking in Louisiana were even higher than national prevalence rates [15] under-reporting and lack of participation by Louisiana in national data monitoring [15,16] could indicate that teen smoking prevalence was even higher than reported. Youth Risk Behavioral Surveillance data showed that 74% of high school students in Louisiana had ever tried cigarettes, and 36% of high school students had smoked a cigarette in the last 30 days (30-day prevalence) [15]. Currently, 25% of high school students in Louisiana smoke and 6,600 individuals under age 18 years became daily smokers each year [17]. The south central area of Louisiana is of particular interest for smoking prevention programs because this area, known as Acadiana, is populated by descendants of French immigrants from Nova Scotia, called “Cajuns,” whose lifestyle includes early initiation of both smoking and drinking behaviors [18]. Unfortunately, Acadiana is an area with “hard-to-reach” adolescent populations and for which very little data have been reported for adolescent smoking.

Good estimates are not available for the number of people in Louisiana who die from causes related to tobacco; however, some 6,400 adults die each year from their own smoking [17]. Consequently, Louisiana ranks high among the 50 states for deaths related to smoking, and its adolescent tobacco-use prevalence is higher than national rates.

National, state and local data have supported significant differences in tobacco use by African American and white adolescents with white students having much higher prevalence rates than black students [9,19]. The population of Louisiana is two-thirds white and one-third African American [20], and local data have shown that there is a lag time between initiation of smoking by white and black adolescents, with white adolescents (males particularly), experimenting in middle school grades and becoming regular smokers in high school and black students initiating smoking in late or post high school [9]. African-American smoking rates continue to increase post high school until they are equal to and/or surpass those for whites at about the late 20s or early 30s.

Middle school smoking prevention programs have been implemented for preventing or delaying the onset of cigarette smoking [21]; however, very few high schools have provided continued support for non-tobacco use throughout the adolescent years. Monitoring the Future (MTF) data indicated that peak ages for initiation of cigarette smoking are in middle school; however, daily smoking developed primarily in grades 8 through 11. Indications are that adolescents who are experimenting in middle school, without peer pressure and environmental supports for not smoking in high school, have a high probability of strengthening the habit, developing nicotine addiction, and becoming part of smoking cliques rather than non-smoking ones. A prevention program in high school can strengthen and perpetuate middle school programs, prevent conversion from experimentation to daily use by white students, and prevent initiation by black students. In Louisiana, however, teacher accountability policies at the state Department of Education level have concentrated all classroom time on preparation for standardized testing with severe penalties for schools that perform below national standards [22]. A prevention program, however, can use the school environment as a conduit for prevention messages and activities, such as student-delivered public service announcements, contests, media, theatrical presentations, and community service.

The Acadiana Coalition of Teens against Tobacco (ACTT) was funded in 2000 with Louisiana MSA funds for the purpose of developing, implementing and evaluating a high school tobacco use prevention/intervention program. The ACTT approach was to use the school environmental opportunities to deliver the intervention. The purpose of the ACTT study was to determine if the intervention could result in a statistically significant difference in 30-day cigarette smoking prevalence between intervention and control schools from baseline to follow-up. Additional outcomes would be examined by race and/or sex.

## 2. Methods

### 2.1. Design

The Acadiana Coalition for Teens against Tobacco (ACTT) was a randomized, controlled cohort study. The cohort was defined as all students enrolled in 9^th^ grade of participating schools at the time of measurement and who completed the ACTT Health Habits Survey (n = 4,763). Twenty-two schools participated from six Louisiana parishes (counties). Twenty schools were research schools and two schools were used for pilot-testing instruments and activities. Schools were stratified by parish and randomized within parish to intervention or control conditions after baseline measurement resulting in ten intervention and ten control schools.

Schools eligible for participation were publicly funded, with no magnet or special populations and within a reasonable proximity to the New Orleans study office. Eight school districts were contacted and six agreed to support the study. One of the six districts contained three high schools, but since only one of them agreed to participate, that school was used as a pilot school. Of the additional 20 schools that were contacted, all agreed to participate.

The principal appointed a school liaison to work with ACTT staff for the purpose of scheduling measurements, setting dates and times for intervention activities, providing logistical support for activities, and providing enrollment lists each semester. The liaison, who received an honorarium, advised ACTT staff about student issues, such as valued incentives, student availability, and information about locations of missing students.

### 2.2. Measurement

#### Health Habits Survey

The Health Habits Survey (HHS) was developed and pilot-tested prior to baseline administration. A full description of the HHS has been published previously [23]. The survey contained a total of 54 items in five sections: 1) demographic information, 2) tobacco use history, 3) alcohol use history, 4) attitudes and beliefs about smoking and drinking, and 5) friends and relatives who use alcohol and tobacco. The primary outcome of ACTT was 30-day prevalence of cigarette smoking. Concordance between three 30-day prevalence questions, each with different wording, was calculated and a high level of agreement between the three questions was found [23]. Baseline measurement occurred in 9^th^ grade, two interim measurements using a modified HHS with only tobacco use history questions was implemented in 10^th^ and 11^th^ grades, and follow-up measurement took place in 12^th^ grade (Table 1). The 12^th^ grade HHS was similar to the baseline survey but some adjustments were made based on student aging.

Students whose parents gave a signed voluntary consent for participation were asked to provide a saliva sample for validation of self-reported smoking within the last 24 hours. Procedures for saliva sampling have been described in detail previously [23]. The samples were analyzed by the Molecular Epidemiology and Biomarkers Research Laboratory, University of Minnesota, Minneapolis [24]. The kappa statistic (κ = 0.69) showed good agreement between responses and cotinine values [23].

#### Data Collection Procedures

For all measurements, except saliva cotinine sampling, students participated with a “passive” consent, that is, parents signed and returned the consent form only if they did not consent to participation. Students also signed an assent form at the time of administration. In most schools, surveys were administered in classrooms. In the remainder of the schools, surveys were administered in assemblies. A second survey administration was scheduled if participation was initially low due to absences, school activities, etc. Saliva cotinine samples were collected at baseline only from those students who had “active” parental consent. These students participated in saliva sampling after they completed the HHS and non-consenting students were dismissed. All data collection forms were identified by a unique 5-digit number randomly assigned before administration and based on enrollment lists provided by the school. All formative, process and outcome data were collected by ACTT staff according to standardized protocols and after practice administrations in the pilot schools.

### 2.3. ACTT Intervention Programs

#### Teacher Workshops

Prior to the beginning of classes each school year, teachers attended an ACTT workshop in which they received current facts about tobacco use and the tobacco companies and were requested to support the ACTT program and encourage student participation in activities. Tobacco use data for the cohort at their school were presented and ACTT staff answered questions and distributed incentives. The first workshop was approximately 40–45 minutes and a teacher tobacco-use survey was administered. In subsequent years, the workshop was no longer than 10–15 minutes and provided teachers with a schedule of ACTT activities for the upcoming year. In the third and final year, a full-color two-sided teacher newsletter was distributed including the data for their students, a calendar of events, and other announcements pertinent to their school.

#### School-Based Media Campaign

The school-based media campaign consisted of posters and Public Service Announcements (PSAs) which were intended to deliver positive modeling and verbal and pictorial persuasion. The media campaign used a specific theme each academic year and these themes determined the media *target* for that year: 1) “Don’t be a sucker!”; 2) “Say No to Big Tobacco”; and 3) a positive send-off message for seniors “Life without Tobacco.” The ACTT logo, “ACTT Smart – Don’t Start” branded all media and other materials for the duration of the intervention. The PSAs were read weekly by students who were known for non-smoking behavior. Message *content* cycled through information transfer (e.g.”We put urea in our cigarettes; urea is found in urine, but it shouldn’t bother you if you can’t taste it, right?” “Happy Valentine’s Day from Big Tobacco),” modeling (e.g. Boy: “Can I have a kiss?” Girl: “Not if you chew tobacco; I’m not kissing a spit-cup.” Boy and Girl: “Kissing is nicer than chewing”), social norms (e.g. “The Real Deal is that most teens don’t smoke – only 13% of you smoked in the last 30 days and 8% smoke regularly”), economics (e.g. “The base of our business is high school students – we need to get you hooked, Sincerely, Lorillard [we make Newports]”), and announcements (e.g.”Join the rest of the country for the Great American Smoke Out; compete against other students and teachers by pledging to stay smoke-free.”). Message *delivery* included rhyme, rap, conversation, and repetition.

The annual poster campaign varied by budget level: low, medium and high [25]. Low budget consisted of posters obtained free of charge from CDC and the American Cancer Society, as well as posters purchased from tobacco prevention websites at minimal charges. Medium budget posters were developed and printed in-house at the university, and a contract with a social marketing firm produced a high budget campaign. Multiple copies of low-budget posters were taped to school walls at specific “life-path points” throughout the school, and multiple copies of medium budget posters were displayed on easels at entry and exit points. Both low and medium budget posters were changed and recycled monthly. The high budget posters were large vinyl posters with grommets that were hung by wires from the ceiling at entry and exit points.

#### Activities

The ACTT program initially was planned as a cohort study. The inability to capture classroom time changed the program to an environmental intervention that targeted the entire student body. Intervention activities are outlined in Tables 2 and 3. Activities were conducted at the general rate of 1–2 per month and were supported by PSAs and posters. The cohort activities, such as the media contest, the introductory quiz and video, and hats off to seniors, were actively delivered to a “captured” target audience and participation rates were high. School wide activities were implemented in the main hallway during lunch periods and were passive in that the students had to initiate contact; therefore, participation rates were much lower when calculated with the entire student body.

#### Parent Newsletter

A full-color two-sided one-page newsletter was distributed to parents once each semester, totaling six over the three-year intervention. The newsletter contained, for example, information about tobacco use and the ACTT program, activity and contest news, and pictures of students engaging in activities. Distribution methods varied with some schools sending the newsletter with report cards and other schools distributing newsletters to students for home delivery.

### 2.4. ACTT Intervention Process Measures

The implementation of the intervention was evaluated for fidelity, exposure and impact, reach, environmental context, and contamination [26]. Fidelity, the extent to which the activities were delivered as intended, was evaluated by observation and a checklist of key components. The evaluation of reach, or participation, was dependent on the particular activity. For classroom presentations (e.g. the introduction and video), reach was an actual count, for hallway activities (e.g. pig lungs, Grossmouth and Jar of Tar), reach was an estimate, and for any activity involving a product (e.g. media contest, psychodrama, advocacy letters), the number of products was counted. Exposure and impact were evaluated for the PSAs and posters at five time points with a self-report student survey. Environmental context included both barriers and facilitators to implementation, e.g. a hurricane and turnover of health educators implementing the program as barriers, and a particularly supportive liaison as a facilitator. Contamination relative to other tobacco control programs or presentations was assessed by principal interview. Graduate students were trained by the health educators and collected the majority of the process data.

### 2.5. Statistical Analyses

The school was the unit of randomization and the unit of analysis for this study. Descriptive statistics (means, medians, and frequencies) were used to summarize the data. Baseline group differences were assessed using Fisher's exact test and t-tests. Prevalence of 30-day smoking, seven-day smoking, and 30-day use of chewing tobacco were computed for each of the 20 schools. Group (control or intervention) and year differences in prevalence were assessed with mixed models repeated measures Analysis of Variance (ANOVA). In another series of analyses, racial and gender composition of the individual schools were incorporated into the models, as was cohort size. The results were essentially the same, so the simpler models were used for interpretation.

## 3. Results

Demographic characteristics of the cohort are shown in Table 4. In both 9^th^ (n = 1,884 *vs* n = 2,575) and 12^th^ grades (n = 1,070 *vs* n = 1,573), there were more students in the control group compared to the intervention group. In 9^th^ grade, the sample was predominantly white with mean age about 15 years for both intervention and control groups. Also, for both intervention and control groups, there were slightly more females than males. In 12^th^ grade, the sample was still predominantly white with mean age about 18 years for both intervention and control groups. Again, there were more females than males.

Characteristics of the schools at baseline are shown in Table 5. In 9^th^ grade the size of the cohort in each school ranged from 55 to 444, with an average school size of 223 (median = 175.5). No significant difference was observed in the average school size between the two groups. Small schools (cohort size below the median) were evenly distributed across the control and intervention conditions. If more than two-thirds of the students in the baseline cohort were white, the school was classified as majority white, and most of the control schools were majority white. At baseline (9^th^ grade), there were no significant differences in the prevalence of tobacco use.

### 3.1. 30-Day Prevalence of Cigarette Smoking

**Figure 1.**
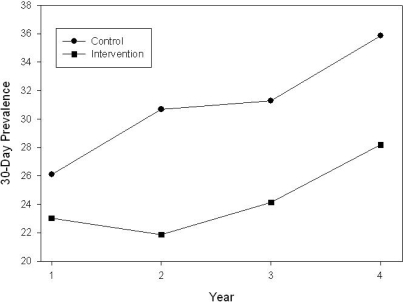
30-day smoking prevalence.

The unit of randomization and the unit of analysis was the school, and the main outcome was 30-day cigarette smoking prevalence. See Table 6 for Prevalence Means and Standard Errors. Figure 1 shows mean 30-day prevalence for intervention and control schools over the four years of the ACTT program. At baseline (9^th^ grade), prevalence was 23.0% for the intervention schools and 26.1% for the control schools. This difference in prevalence between intervention and control schools at baseline was non-significant. It should be noted that no intervention activities were conducted during this first baseline year. Intervention activities began during the fall semester of 10^th^ grade when prevalence in the intervention schools decreased to 21.9% and increased in the control schools to 30.7%. During the next intervention year in 11^th^ grade, however, prevalence increased to 24.1% in the intervention schools, representing a 1.1% increase over baseline, while prevalence continued to increase in the control schools to 31.3%, representing a 5.2% increase over baseline. By the 12^th^ and final year of the intervention, the difference in 30-day prevalence between the intervention and control schools was 7.0%, 27.3% in the intervention schools and 34.3% in the control schools. The time x treatment interaction was not statistically significant, p = 0.40).

### 3.2. 7-Day Prevalence of Cigarette Smoking

**Figure 2.**
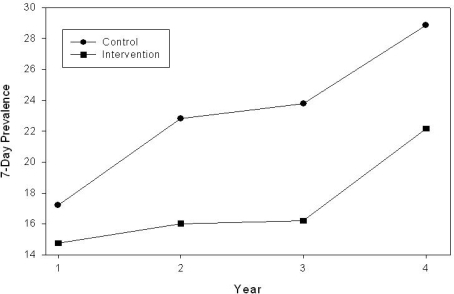
7-day smoking prevalence.

Similar results were seen for 7-day prevalence, as shown in Figure 2. In Year 1, 7-day smoking prevalence in intervention *vs* control schools was 14.8% *vs* 17.2, respectively. In Year 2 a differential increase of 1.2% was observed in intervention schools and 5.6% in control schools, making a total difference between the two groups of 6.8%. By Year 3, the difference between intervention and control schools had increased to 7.6%, with intervention schools at 16.2% and control schools at 23.8%. By the fourth and final year, 7-day prevalence in the intervention schools *vs* the control schools represented increases of 5.3% *vs* 3.3%, respectively. Overall, the increase in prevalence from year 1 to year 4 was 9.9% for control schools and 6.4% for intervention schools. The time x treatment interaction was not statistically significant, p = 0.36.

### 3.3. 30-Day Prevalence for Smokeless Tobacco

**Figure 3.**
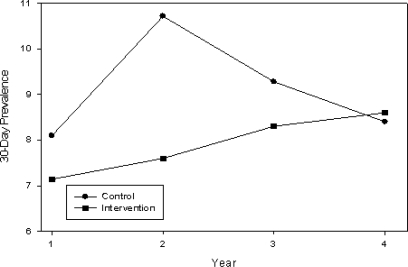
30-day chewing tobacco prevalence.

The pattern of 30-day prevalence for smokeless tobacco was different from the pattern for smoking prevalence (Figure 3). From Year 1 to Year 2 a small increase of 0.5% in prevalence was observed in intervention schools and a larger increase was observed in control schools. Prevalence in the intervention schools continued to increase during Years 3 and 4, respectively; however, prevalence decreased during the same years for the control schools. The total increase in prevalence from baseline to follow-up in the intervention schools was 1.5% and in the control schools was 0.3%, resulting in a crossover of data for intervention and control schools in Year 4. There was generally a low prevalence (< 11%) of chewing tobacco use and most users were white males [23]; however, there was no significant time x treatment interaction, p = 0.13. In summary, for 30-day and 7-day smoking prevalence and 30-day prevalence of smokeless tobacco, there were no statistically significant differences between groups at baseline. There were no statistically significant time x treatment interactions for the three kinds of prevalence examined.

### 3.4. 30-Day Prevalence by Individual Schools

Table 7 presents the data for 30-day prevalence of cigarette smoking and percent white students in 9^th^ and 12^th^ grades at participating schools. Only five schools demonstrated a decline in prevalence over the four years of the study, with four of those in the intervention condition and one in the control condition. It should be noted that all of the schools showing a decreased prevalence were majority African-American. No decreases in prevalence were observed in any of the majority white schools.

## 4. Discussion

The 25% overall 30-day cigarette smoking prevalence at baseline was higher than was reported nationally for 9^th^ graders [15]. The Youth Risk Behavior Surveillance (YRBS) [27] reported an overall 30-day prevalence of 23.9% nationally, while The Monitoring the Future Study (MFS) [19] reported 30-day prevalence of 12.2% for 8^th^ graders 21.3% for 10^th^ graders. Obviously, a strong rationale existed for conducting a tobacco control program in Louisiana. At follow-up, when the cohort was in the 12^th^ grade, 30-day prevalence in the intervention schools was 27.3%, still higher than the 25% for 12^th^ graders reported by MFS and 23% reported by the YRBSS. It is clear that a larger difference in 30-day prevalence existed between intervention and control schools in 12^th^ grade compared to 9^th^ grade; however, no statistically significant interaction between treatment condition and time was observed, resulting in a non-significant intervention effect. Similarly, no significant intervention effects were observed for 30-day smokeless tobacco use, where 12^th^ grade prevalence was consistent with cross-sectional national data [19]. Also consistent with national data was that more boys used smokeless tobacco than girls [19,27].

### 4.1. Study Design

To understand the absence of significant effects, it is useful to review the study design. The unit of randomization and, therefore, the unit of analysis, was the school, rather than the individual. This design is well accepted as the gold standard by school-based randomized controlled trials conducted in the United States, whether they are conducted at a single site or at multiple sites [28–30]. This is the most rigorous design for statistical consideration of variability between school sites. Our power analysis had indicated that a total of 20 schools would be sufficient to detect a statistically significant difference between intervention and control schools over the time course of the intervention; however, this was not the result observed. Study power, of course, was based on equivalent data at baseline, and, although not statistically different, there were differences in the primary outcome (30-day smoking prevalence) between intervention and control schools at baseline. We have two options to consider, therefore: one is that we did not have sufficient power, with the school as the unit of analysis, to achieve significance for the difference in prevalence observed at follow-up in 12^th^ grade; and the other option is that we could have used a different randomization scheme. The 20 study schools were located within five school districts. It was considered politically correct to stratify by school district and randomize within district. Randomizing schools without stratification may have achieved better equivalence at baseline; however, the chance that some school districts would have all control schools would have made implementation of the program in those districts difficult. We can have some confidence, then, that the study design, the power analysis conducted prior to the study, and the randomization scheme were appropriate based on the information available.

### 4.2. The Intervention

A cohort was identified for the purpose of longitudinal evaluation of the study. It was recognized that academic benchmarks and teacher accountability programs developed at the state level and evaluated by Louisiana Educational Assessment Program (LEAP) and other standardized tests would limit availability of class time. The intervention was then designed as a school-based environmental intervention with strategies focused on a media campaign (posters and public service announcements) [25], lunch-time activities conducted at major student access points, and student activism in support of state legislation for tobacco control. Some of the advantages of the environmental program compared to a classroom-based program were: exposure of the entire student body to the intervention, easier and more flexible scheduling of activities, reduced staff time for contacting individual teachers for scheduling, and decreased cancellation of scheduled activities. These advantages, however, may have been overshadowed by some of the challenges of the environmental program, which were: low participation rates in hallway activities, weak program dose, lack of program recognition, weakened budget impacting the value of student incentives, and decreased importance in the school routine.

There is evidence supporting attendance at intervention sessions and/or participation in intervention activities for achieving behavior change [31]. Classroom-based intervention activities would have had a captive audience. Process evaluation of activities conducted during the first intervention semester showed that attendance at those activities conducted in the classroom had greater than 90% participation. Evaluation conducted in the following semester showed a decline in prevalence rates in intervention compared to control schools. Once activities moved to hallways and became lunch-time activities, process evaluation estimates of reach (participation) were generally low for large schools (greater than 1100 students) and moderate for smaller schools (less than 800 students). Participation in these activities required active initiation by students along with relinquishing lunch time to participate in the activities. Although process evaluation could not document this, it is assumed that those students who were already smoking were least likely to actively engage in these intervention activities. Consequently, many students who would have benefitted the most from the intervention may have been missed. In addition, while process evaluation provided participation estimates, it could not document who was participating. There was no evidence, therefore, that the students in the cohort (the only students who were measured) and/or the students who were already smoking were being reached by the intervention. Consequently, documented low participation rates along with the probability that the students for whom the program was intended may not be participating, would have resulted in a low program dose for those students.

Because program exposure included the entire student body and not just the cohort, the incentive budget, originally estimated for the cohort only, was stretched thin. This resulted in giveaways and incentives that were of much less value than intended and may not have been of sufficient value to encourage students to participate.

Recognizing these challenges with the intervention activities, it was realized that the environmental media campaign was of much greater importance than originally realized. The media campaign was evaluated by Hong *et al*. (2008), and found to have satisfactory exposure among students [25], along with some preventative and behavior change effects. For example, consistently across the three years of the intervention, about one-third of students reported that the posters helped prevent them from smoking, while about 10% of students reported that the posters helped them to quit smoking. Initially, the public service announcements were observed not to have as high exposure as the posters; however, as the program became better known, this exposure increased.

Another process evaluation variable that was informative regarding the implementation of the intervention were documented “contextual” factors or events that affected the implementation of the ACTT program over which the program had no control. For example, two of the intervention schools were destroyed by tornadoes accompanying and following Hurricane Lily. These schools, of course, lost all of their environmental media and students resumed classes by sharing space in schools not participating in the ACTT program. Media, therefore, could not be implemented in those schools. Two other intervention schools were found not to have exhibited the posters for at least two semesters.

A possible confounding variable was school district smoking policy [32]. At the time of the study, four of the five participating school districts had a “restricted” smoking policy, i.e. adults could smoke in designated places on campus. Only one of the school districts had a no tobacco use policy which prohibited smoking anywhere on the school premises. Four of the 20 study schools were located in this district, two intervention and two control schools. It should be noted that of the five schools that demonstrated a decline in 30-day prevalence between baseline and follow-up, three schools were in the district with a no tobacco use policy.

## 5. Conclusions

We have confidence that the study design was strong and the randomization scheme appropriate. Consequently, the intervention effect size was not strong enough to achieve statistical significance, and this has been the result of many school-based education type tobacco control programs [33]. Process evaluation data showed that several factors could have impacted intervention results. Variables such as low reach or participation, inconsistent exposure to media, weak program dose, and contextual factors beyond the control of the program staff influenced program implementation. The school-based environmental strategy had some advantages; however, challenges, such as dilution of the focus of the intervention, probably overcame any potential advantages. With only the cohort students being measured and evaluated, and, without documentation that the cohort was actually being reached sufficiently by the program, valuable lessons have been learned that need to be addressed by, for example, the Centers for Disease Control and Prevention (CDC) in developing priority guidelines for school-based smoking prevention programs [34]. With statewide school accountability systems in place, especially in high schools, it is no longer realistic to expect class time for a tobacco control curriculum. Instead, we need to focus on developing stronger and more feasible environmental programs that will reach as many students as possible. The ACTT media campaign, for example, was moderately successful, and a stronger focus on presentation of media through the use of social marketing principles would contribute strongly to any environmental tobacco control program [35].

In addition, a no-smoking policy with clear and published guidelines for enforcement could provide a relevant and important environmental support for the no-smoking message [36].

## Figures and Tables

**Table 1.** ACTT Measurement Schedule in 20 Schools over Four Years.

**Cohort Grade Level**	**Semester**	**Measurement**
9^th^	Fall, 2000	Formative focus groups
9^th^	Spring, 2001	Baseline Health Habits Survey and saliva cotinine sampling
10^th^	Spring, 2002	Interim Modified Health Habits Survey*
11^th^	Spring, 2003	Modified Health Habits Survey*
12^th^	Spring, 2004	Follow-up Health Habits Survey Exit focus groups

* Modified Health Habits Survey = tobacco use questions only.

**Table 2.** ACTT Intervention Schedule in 10 Schools over Three Years.

**Cohort Grade Level**	**Semester**	**Intervention Activities**	
		**Cohort (Classroom) Only**	**Schoolwide (Hallway)**
10^th^	Fall 2001	Introduction to program & video Media contest	“Don’t be a sucker” interactive exhibit Great American SmokeOut Teacher workshop PSA’s (weekly) Posters (monthly)
10^th^	Spring 2002		Kick Butts Day PSA’s (weekly) Posters (monthly)
11^th^	Fall 2002		Mr. Grossmouth/Jar of Tar Great American SmokeOut PSA's (weekly) Poster (semester)
11^th^	Spring 2003	Psychodrama	Advocacy Big Tobacco Valentine's Day cards Kick Butts Day (with pig lungs) PSA's (weekly) Posters (bimonthly)
12^th^	Fall 2003	Hats off to seniors	Smoking Roulette Great American SmokeOut PSA's (weekly) Posters (monthly)
12^th^	Spring 2004		Can you handle the TRUTH? Tobacco Jeopardy Kick Butts Day PSA's (weekly) Posters (monthly)

**Table 3.** ACTT Intervention Activities.

**Activities**	**Description – Cohort Activities**	**Semester(s)**
Introduction & video	Introduction to ACTT; interactive quiz about tobacco with questions and prizes. A video “Unfiltered” featured a popular MTV Real World star on a quit smoking weekend with other teens.	Fall, 2001
Media Contest	Cohort volunteered to develop any type of media with an anti-smoking message. Prizes awarded.	Fall, 2001
Valentine Day cards	Four different Valentine Day cards tongue-in-cheek messages from Big Tobacco	Spring, 2003
Psychodrama	Students competed for trophies by developing, implementing and videotaping a dramatic skit with a smoking prevention theme.	Spring, 2003
Hats off to seniors	At an ACTT-sponsored breakfast or lunch, seniors were honored and thanked for participating in the program. Slides of activities at their school were shown on a large screen and table tents contained smoke-free messages.	Fall, 2003
	**Description – Schoolwide Activities**	
Grossmouth/ Jar of Tar	A chewing tobacco quiz with true/false questions focusing on a display of a model of a diseased mouth and jar with dark liquid representing tar in lungs after smoking one year.	Fall, 2002
Legislative advocacy	Students sent letters to their state senators requesting the repeal of the tobacco preemption law. The law was repealed and the announcement was made in the intervention schools.	Spring, 2003
Smoker’s roulette	Students spun a roulette wheel to identify categories of questions, and, if answered correctly, won prizes.	Fall, 2003
Can you handle the TRUTH?	Can you handle the truth about Big Tobacco messages were placed in strategic places throughout the school. Students wrote anti-smoking messages on banners to be sent to tobacco companies.	Spring, 2004
Jeopardy	Patterned after the TV game, students competed by responding to tobacco facts with a question.	Spring, 2004
Pig lung demonstration	Diseased pig lungs representing a 10-year smoker were touched and examined by students.	Spring 2002, 2003
What’s in a cigarette?	Competitive scavenger hunt for the names of 60 chemicals found in cigarettes which were placed individually around the school.	Spring 2002
Great American SmokeOut	National observance in which pledges not to smoke or to stop smoking were signed by students and teachers and taped to school walls. The grade level with the most pledges won a prize awarded by the school.	Fall, 2001, 2002, 2003
Kick Butts Day	Students asked to join with students nationally to kick butts and participated in various activities.	Spring, 2002, 2003, 2004

**Table 4.** Demographic Characteristics of the ACTT Cohort for 9^th^ and 12^th^ Grades.

**Demographic Characteristics**	**9^th^ Grade**			**12^th^ Grade**		
	**Intervention (%)**	**Control (%)**	**Total (%)**	**Intervention (%)**	**Control (%)**	**Total (%)**
Race/Ethnicity White African-American	1,133 (60) 751 (40)	1,770 (69) 805 (31)	2,903 (65) 1,556 (35)	668 (62) 402 (38)	1,144 (73) 429 (27)	1,812 (69) 831 (31)
Total n	1,884	2,575	4,459	1,070	1,573	2,643
Sex Male Female	907 (48) 975 (52)	1,255 (49) 1,317 (51)	2,162 (49) 2,292 (51)	456 (43) 613 (57)	696 (44) 874 (56)	1,152 (44) 1,487 (56)
Total n	1,882	2,572	4,454	1,069	1,570	2,639
Age = Mean Years Range	15.4 14.0–18.5	15.3 11.8–19.0	15.4 11.8–19.3	18.1 15.6–21.2	18.1 14.4–20.4	18.1 14.4–21.2

ACTT = Acadiana Coalition of Teens against Tobacco

**Table 5.** Baseline Characteristics of ACTT Study Schools.

	**Condition**	
	**Control (n = 10)**	**Intervention (n = 10)**
Size of cohort Mean + SD Small (≤ 175)	258 + 153 4	189 + 136 6
% Male	48.5 + 5.2	49.6 + 5.1
Majority white (> 66.7%)	7	4
Smoking 30 day Prevalence 7 day Prevalence	26.4 + 4.2 17.2 + 3.0	23.0 + 7.0 14.8 + 5.2
Chewing Tobacco 30 day Prevalence	8.1 + 3.8	7.1 + 3.8

**Table 6.** Means and Standard Errors (SEs) by Study Group for the ACTT Cohort.

	Group		
	Control (n = 10)	Intervention (n = 10)	
Year	Mean ± SE	Mean ± SE	F and p values
30-Day Smoking Prevalence			
1 2 3 4	26.1 ± 1.3 30.7 ± 2.5 31.3 ± 3.2 34.3 ± 3.3	23.0 ± 2.2 21.9 ± 1.9 24.1 ± 2.5 27.3 ± 3.2	
Year 1 to Year 4 Prevalence Rate	8.2 ± 2.9	4.3 ± 4.2	F_3,54_ = 0.99, p = 0.40
7-Day Smoking Prevalence			
1 2 3 4	17.2 ± 0.9 22.8 ± 2.1 23.8 ± 2.8 27.1 ± 3.2	14.8 ± 1.7 16.0 ± 1.5 16.2 ± 2.2 21.5 ± 2.6	
Year 1 to Year 4 Prevalence Rate	9.9 ± 3.1	6.7 ± 3.5	F_3,54_ = 1.09, p = 0.36
30-Day Smokeless Tobacco Prevalence			
1 2 3 4	8.1 ± 1.2 10.7 ± 1.3 9.3 ± 1.1 8.4 ± 1.0	7.1 ± 1.2 7.6 ± 0.9 8.3 ± 1.2 8.6 ± 1.3	
Year 1 to Year 4 Prevalence Rate	0.3 ± 1.3	1.5 ± 1.0	F_3,54_ = 1.93, p = 0.13

**Table 7.** 30-Day Smoking Prevalence for the ACTT Cohort at 9^th^ and 12^th^ grades with Group Assignment, and Percent of White Students.

**Group Assignment**	**Grade**			
	**9th**		**12th**	
	**% Prevalence**	**% White**	**% Prevalence**	**% White**
1	26.5	97.1	43.6	98.2
1	32.2	73.1	33.9	74.7
1	24.4	73.0	29.3	74.1
1	21.2	56.0	31.3	62.6
1	28.7	96.2	50.5	97.4
1	29.8	61.5	29.5	66.1
1	20.7	27.5	15.2	25.3
1	20.2	87.3	32.7	90.9
1	29.5	92.9	43.0	93.0
1	27.9	92.3	34.9	90.7
2	25.2	19.1	17.7	16.1
2	12.7	80.0	50.0	71.0
2	19.6	50.9	21.2	54.6
2	26.1	82.5	34.5	88.0
2	13.7	22.7	12.2	20.9
2	25.1	80.0	32.6	82.9
2	29.5	22.9	22.2	16.7
2	33.9	33.0	28.6	32.9
2	16.5	58.7	28.4	67.6
2	27.7	94.1	33.3	92.7

1 = Control; 2 = Intervention
